# Cattle mitogenome variation reveals a post-glacial expansion of haplogroup P and an early incorporation into northeast Asian domestic herds

**DOI:** 10.1038/s41598-020-78040-8

**Published:** 2020-11-30

**Authors:** Hideyuki Mannen, Takahiro Yonezawa, Kako Murata, Aoi Noda, Fuki Kawaguchi, Shinji Sasazaki, Anna Olivieri, Alessandro Achilli, Antonio Torroni

**Affiliations:** 1 Laboratory of Animal Breeding and Genetics, Graduate School of Agricultural Science, Kobe University, Kobe, Japan; 2 Faculty of Agriculture, Tokyo University of Agriculture, Atsugi, Japan; 3 Dipartimento di Biologia e Biotecnologie “L. Spallanzani”, Università di Pavia, Pavia, Italy

**Keywords:** Evolutionary genetics, Molecular evolution, Phylogenetics, Population genetics

## Abstract

Surveys of mitochondrial DNA (mtDNA) variation have shown that worldwide domestic cattle are characterized by just a few major haplogroups. Two, T and I, are common and characterize *Bos taurus* and *Bos indicus*, respectively, while the other three, P, Q and R, are rare and are found only in taurine breeds. Haplogroup P is typical of extinct European aurochs, while intriguingly modern P mtDNAs have only been found in northeast Asian cattle. These Asian P mtDNAs are extremely rare with the exception of the Japanese Shorthorn breed, where they reach a frequency of 45.9%. To shed light on the origin of this haplogroup in northeast Asian cattle, we completely sequenced 14 Japanese Shorthorn mitogenomes belonging to haplogroup P. Phylogenetic and Bayesian analyses revealed: (1) a post-glacial expansion of aurochs carrying haplogroup P from Europe to Asia; (2) that all Asian P mtDNAs belong to a single sub-haplogroup (P1a), so far never detected in either European or Asian aurochs remains, which was incorporated into domestic cattle of continental northeastern Asia possibly ~ 3700 years ago; and (3) that haplogroup P1a mtDNAs found in the Japanese Shorthorn breed probably reached Japan about 650 years ago from Mongolia/Russia, in agreement with historical evidence.

## Introduction

All modern cattle derive from wild ancestral aurochs (*Bos primigenius*), which were distributed throughout large parts of Eurasia and Northern Africa during the Pleistocene and the early Holocene^1,2^. Modern *Bos taurus* and *Bos indicus* are the result of two independent domestication events; the first occurred 10,000–11,000 years ago in the Upper Euphrates Valley, the second about 2000 years later in the Indus Valley^3–6^. Surveys of mitochondrial DNA (mtDNA) variation in modern cattle revealed one well-diverged major haplogroup in *B. taurus* (haplogroup T) and one in *B. indicus* (haplogroup I) as well as the much rarer haplogroups P, Q and R^7^. In contrast, haplogroup C and E mtDNAs have been detected only in ancient samples^8–11^.

Haplogroup P was the most common in European aurochs according to ancient DNA surveys^8,12,13^, but to date it has not been observed in modern European cattle. However, it has been detected in three modern Asian specimens (two from Korea and one from China)^14,15^, although at an extremely low frequency taking into account that thousands of cattle samples have been surveyed for mtDNA variation^7,16–19^. These observations were initially interpreted as indicating that the presence of haplogroup P mtDNAs in modern specimens was due to a limited amount of introgression from wild aurochs into early domesticated herds^9,14^. However, we recently discovered, by surveying the entire mtDNA control-region, that haplogroup P harbors an unexpected high frequency, 83 out of 181 samples, in Japanese Shorthorn cattle^20^. We also detected its presence, but at a very low frequency (2 out of 105) in Japanese Holstein cattle from northern Japan^21^. These observations raise the possibility that haplogroup P mtDNAs might represent the legacy in some modern northeast Asian cattle breeds of a minor and local event of domestication/introgression of Asian aurochs.

Previous studies have shown that analyses of entire mitogenome sequences are much more informative than mtDNA control-region surveys and better suited to reveal the fine phylogenetic structure of the cattle mtDNA tree and to estimate haplogroup coalescence times^22–24^. Therefore, here we extended to the highest level of molecular resolution, i.e. entire mitogenome sequences, our analyses of haplogroup P mtDNAs from Japanese Shorthorn cattle by completely sequencing 14 mitogenomes. Phylogenetic and Bayesian analyses of the novel haplogroup P mitogenomes together with those previously published reveal a post-glacial expansion of aurochs carrying haplogroup P from Europe to Asia and a relatively recent introgression of their mitogenomes into some domestic herds that most likely lived in continental northeastern Asia.

## Results

### Sequence variation of bovine haplogroup P mitogenomes

To shed light on the origin of haplogroup P in modern northeast Asian cattle, we sequenced the entire mitogenome from 14 Japanese Shorthorn samples previously classified within haplogroup P. This allowed the detection of ten distinct haplotypes (JS1-JS10) (DDBJ accession numbers: LC537308 - LC537317). Two of the ten haplotypes, JS1 and JS3, were represented four and two times, respectively (Supplementary Fig. S1). The sequence alignment of the Japanese Shorthorn P mitogenomes along the other three previously published (DQ124389, GU985279 and JQ437479) and the bovine reference sequence (V00654) revealed 99 variant sites including four indels (Supplementary Fig. S1a), whereas a sequence comparison limited only to the mitogenomes belonging to haplogroup P revealed 44 variants including two indels (Supplementary Fig. S1b).

### Phylogenetic analyses of bovine haplogroup P using entire mitogenomes

**Figure 1:**
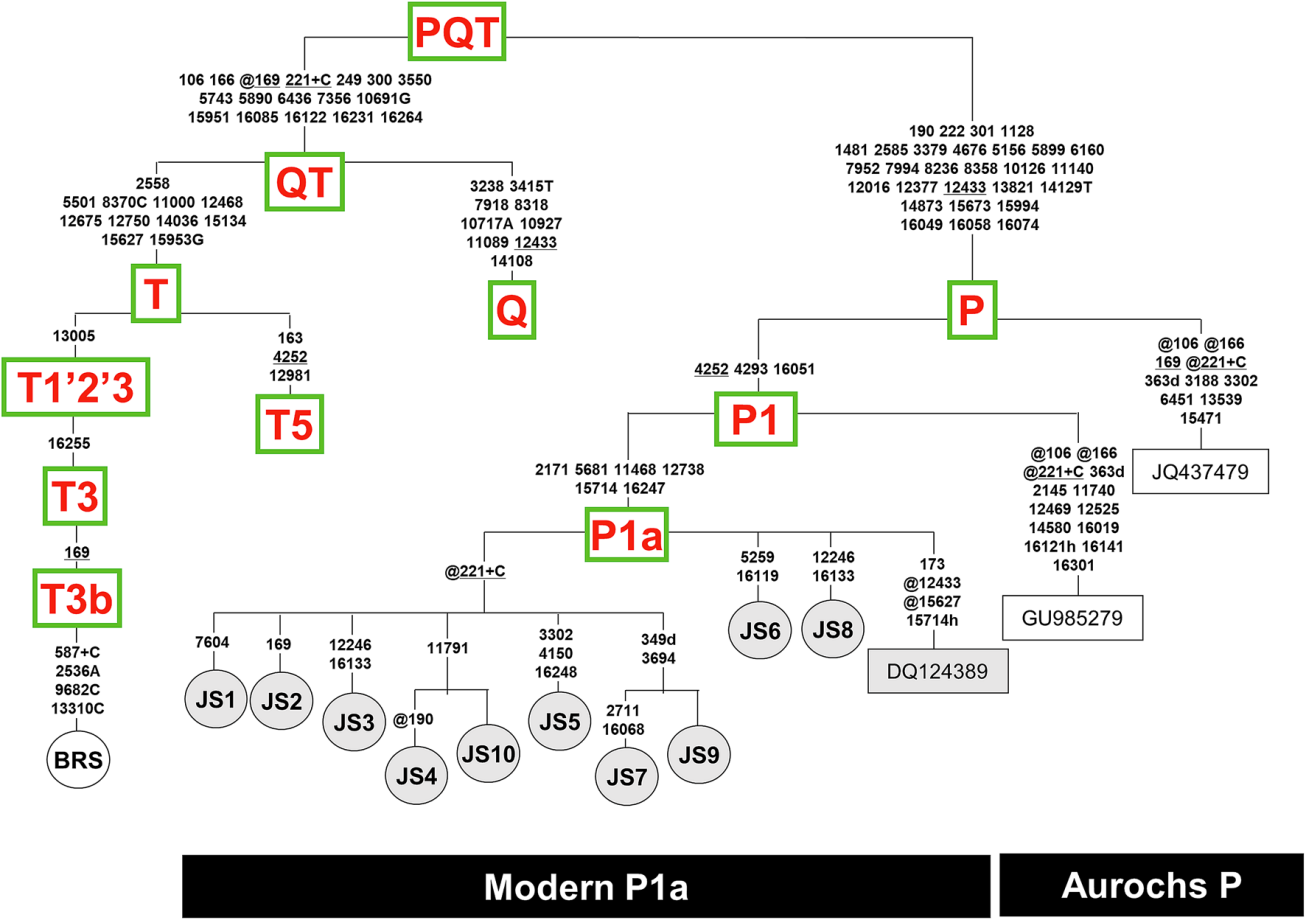
Schematic phylogenetic tree of cattle mtDNA haplogroups encompassing all available mitogenomes belonging to haplogroup P. Haplogroup P mitogenome sequences JS1–JS10 have been determined in this study; JS1 and JS3 were detected in four and two Japanese Shorthorn specimens, respectively, while the others were observed in a single specimen. Their GenBank accession numbers are reported in Supplementary Fig. S1. Sequences DQ124389 (Korea), GU985279 (England) and JQ437479 (Poland) are from public databases. This tree was built as previously described^7,14,22–24^. Mutations are shown on the branches and are numbered according to the Bovine Reference Sequence (BRS) (V00654); they are transitions relative to the BRS unless a base is explicitly indicated; suffixes indicate transversions (to A, G, C, or T) or indels (+ , d) and heteroplasmy (h). Recurrent mutations within the phylogeny are underlined and back mutations are marked with the prefix @. Note that the reconstruction of recurrent mutations in the control region is ambiguous in a number of cases. Detail information of the variants is indicated in Supplementary Fig. S1.

The phylogenetic relationships of the novel and previously published P mitogenomes as well as representative mitogenomes belonging to other haplogroups are illustrated in Fig. 1 and Supplementary Fig. S2. The ten Japanese Shorthorn haplotypes (JS1-JS10) form a star-like sub-cluster defined by six transitions (nps 2171, 5681, 11,468, 12,738, 15,714 and 16,247) that includes also the previously reported mitogenome from Korea (DQ124389). This well-defined sub-cluster, which we named P1a (Fig. 1) according to current haplogroup nomenclature, corresponds to the sub-haplogroup Pc previously identified solely on the basis of the control-region transition at np 16,247^20^. The two remaining P mitogenomes are from European aurochs (JQ437479 and GU985279 from Poland and England, respectively) and branch off earlier in the phylogeny of P, suggesting Europe as the most likely homeland for haplogroup P.

To further assess the issue of the origin of Asian P mtDNAs, we built a phylogenetic tree based on 410-bp long control-region sequences (nps 15,903–16,312). This allowed the inclusion of many previously published P mtDNAs whose sequence variation had been surveyed only in that region (Supplementary Fig. S3)^8,20,21^. The control-region phylogeny supports the scenario that the cluster harboring the mutation at np 16,247 (P1a) is northeast Asian-specific (only mtDNAs from Japan, Korea and China) while all P mtDNAs from European aurochs cluster into a separate branch that we named “aurochs P” (Fig. 1 and Supplementary Fig. S3).

### Haplogroup P coalescence times based on entire mitogenome sequences

ML coalescence times were estimated by using all or 3rd codon substitutions of mtDNA protein-coding genes (Supplementary Table S1). The ML age estimates of the most recent common ancestor for each of the major haplogroups are generally consistent with those estimated in previous studies^7,14,23^. Time estimates for haplogroup P (node 7) and the P1a cluster (node 9) are, respectively, 16,180 ± 4560 and 3720 ± 1770 years before present (YBP) when using all substitutions in protein-coding genes, and 8770 ± 3620 and 2900 ± 1980 YBP when using only substitutions in the 3rd codon position (Supplementary Table S1).

### Estimating past demographic trends for haplogroup P

**Figure 2:**
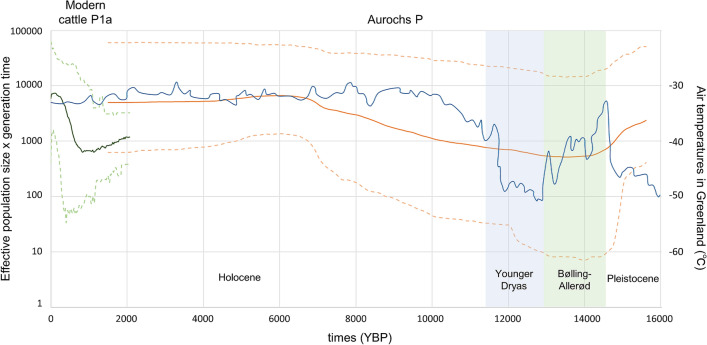
Bayesian skyline plot showing population size trend for haplogroup P and temperature changes in Greenland. The Y axis indicates the effective number of female × generation times (left side), as inferred from the control-region dataset of haplogroup P mtDNAs and temperature changes in Greenland (right side). The solid line is the median estimate while the broken lines indicate the 95% highest posterior density limits. The green and orange lines are the estimates for P1a and aurochs P (excluding P1a mtDNAs), respectively. The blue line indicates temperatures in the post-glacial period according to Greenland ice cores^25^.

A Bayesian skyline plot (BSP) was constructed using available 410-bp long control-region sequences from P mtDNAs^8,20^ in order to increase the sample size. The BSP of haplogroup P shows five major changes in the effective female population size, three in aurochs P (excluding P1a mtDNAs) and two in modern P1a (Fig. 2). Figure 2 also shows estimated temperatures in the post-glacial period according to Greenland ice cores^25^. When using aurochs P mtDNAs, the effective population size shows a rather sharp reduction between 16,000 and 13,500 YBP, then a gradual increase between 13,500 and 6000 YBP, followed by a slight reduction between 6000 and 1500 YBP. The demographic fluctuation of aurochs may relate to the drastic temperature changes that occurred during the Bølling–Allerød (14,700 to 12,700 YBP)^26^ and the Younger Dryas (12,800 to 11,550 YBP)^27^, and overall appears to coincide with temperature changes. As for modern cattle sub-haplogroup P1a, the population size shows a reduction between 2000 and 1400 YBP and a sharp increase starting around 650 YBP. This recent and sharp increase explains the star-like topology of P1a and resembles those previously reported for domestic cattle and other bovine domestic species^28^.

## Discussion

The phylogenetic analyses carried out in this study allowed for the first time to define, at the highest level of molecular resolution, the internal structure of cattle haplogroup P and the relationships of some of its modern and past members (Fig. 1 and Supplementary Fig. S2). The 14 novel P mitogenomes from Japanese Shorthorn cattle, a breed that harbors an extremely high frequency of this haplogroup (45.9%)^20^, were all found to cluster within a well-defined and derived sub-haplogroup of P that we termed P1a (Fig. 1). This sub-haplogroup harbors a star-like structure and includes also the Korean mitogenome DQ124389, the only complete P mitogenome from Asia. The remaining two complete P mitogenomes, both from extinct European aurochs, branch off earlier in the phylogeny of P and from two distinct nodes, suggesting that the ancestral source of P was most likely Europe. Among the six transitions (Fig. 1) that define sub-haplogroup P1a, the one at np 16,247 is located in a stretch of the control-region that has been surveyed in previous studies of P mtDNAs from extinct European aurochs^8,15^ and modern Japanese cattle^20,21^. This mutation characterizes all previously identified P mtDNAs from Japan (N = 85) as well as all other P mtDNA control-regions reported in Asia, one from China (AY998840) and one from Korea (AY337527), but it is absent in all P mtDNAs from European aurochs. In brief, sub-haplogroup P1a appears to be restricted to Asia and the only branch of P present in Asia.

Coalescence time estimates for the nodes of P, P1 and P1a are 16,180–8770 YBP, 12,530–8770 YBP and 3720–2900 YBP, respectively (Supplementary Table S1), time ranges that include estimates obtained from both whole codon and only third codon positions. Our age estimate for P is overall consistent with that previously proposed^12^, and that for P1 is in line with the radiocarbon date (6738 ± 668 calibrated YBP) of the humerus bone found in a cave in Derbyshire^12^ from which derives the only P mitogenome departing from the P1 node (Fig. 1).

In contrast to haplogroups T and Q, haplogroup P did not undergo domestication, thus its coalescence age reflects an expansion event of the extinct wild aurochs population(s) that harbored this haplogroup, an expansion which in turn was most likely triggered by a change in climate conditions.

**Figure 3:**
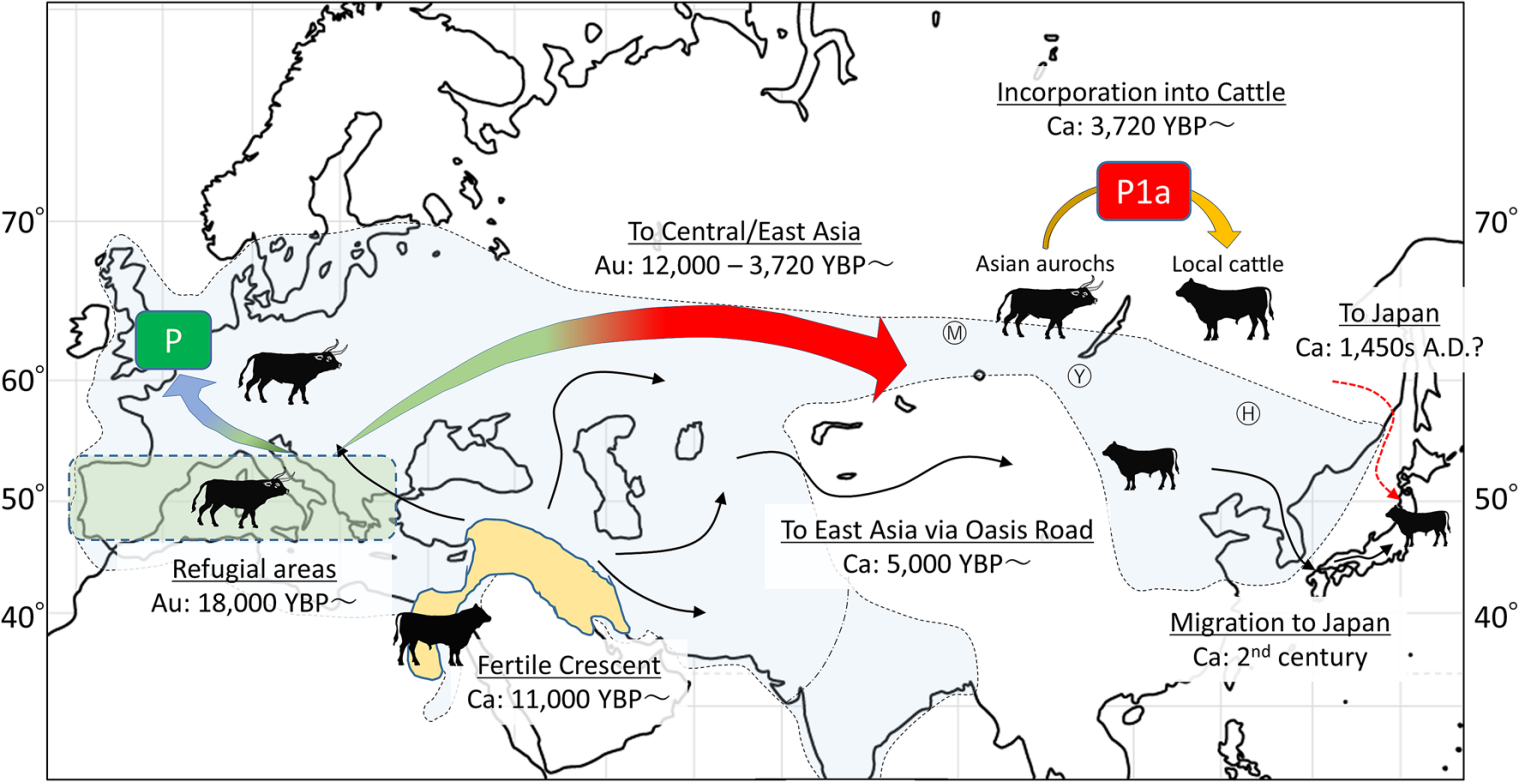
A model for the geographical and temporal spread of haplogroup P. Map showing divergence and spread routes hypothesized in this study for haplogroup P. Colored arrows indicate possible movements of wild aurochs with haplogroup P mtDNAs. Solid-line arrows indicate generally well accepted domestication routes of cattle from the Fertile Crescent^2^. The red broken-line arrow indicates the possible migration route of haplogroup P to Japan. The dates on the map were estimated by previous studies (see text). Au: aurochs events. Ca: domesticated cattle events. The light blue area enclosed by the broken line corresponds to the distribution range of the aurochs. The outer most border of the range was during the Pleistocene^2^. Ⓗ: Houtaomuga ruins in northeast China. Ⓜ: Minusinsk ruins in the South Siberia. Ⓨ: Southern Baikal, the place of origin of the Yakutian breed, the last remaining Siberian native cattle. The map was generated using Microsoft PowerPoint 2019 based on a map by 3kaku-K (https://www.freemap.jp/item/world/world1.html).

During the Last Glacial Maximum (LGM) (23–18 kYBP), the European ice sheet extended south to 52nd parallel north and permafrost south to the 47nd^29,30^. The distribution range of European aurochs during the LGM possibly included Southern France, Italy and the Balkans, with the Iberian and Italian peninsulas acting as refugial areas^13^. As the ice melted, most temperate species, including aurochs, diffused from the refugial areas^29^. The estimated divergence times between the P and P1 (16,180–8770 YBP), and between P1 and P1a (12,530–8770 YBP) matches well with the early post-glacial period, suggesting that the divergence of P1a from P and P1 occurred in this period after the LGM (Fig. 3). This is roughly the same time frame in which aurochs P shows a substantial population size increase in the BSP (Fig. 2).

Figure 3 illustrates a model for the geographical and temporal spread of haplogroup P from Europe, its most likely ancestral homeland, to Japan. In such a scenario, after its origin in Europe, the diffusion of haplogroup P to Central/East Asia and its concomitant and gradual differentiation into P1a would have occurred via Kazakhstan/Russia-China/Mongolia-China northerly route (light blue area in Fig. 3) to avoid the Himalaya Mountains and the Tibetan Tableland and the associated cold and dry climate conditions. At Minusinsk, an archeological site of South Siberia (M symbol in Fig. 3), aurochs, bears and elks are depicted in rock paintings from the Neolithic to the Bronze Age^31^.

As for domestic cattle, archeological and molecular evidence have shown that taurine cattle (*B. taurus*) first appeared in North China in the late Neolithic between approximately 5000 and 3000 YBP^32^. Our time estimate for the P1a node is 3720–2900 YBP, thus a bit younger than the above mentioned arrival time of cattle in East Asia. This raises the possibility that P1a mtDNAs, seen sporadically in Chinese and Korean cattle, and at high frequency in Japanese Shorthorn cattle, might represent the legacy of a domestication/introgression event of aurochs cows harboring haplogroup P1a into one of the early local domestic herd in Central/East Asia (Fig. 3).

When trying to unravel the origin of P1a in modern northeast Asian cattle some mtDNA data should be also taken into consideration. Yakutian cattle are the last remaining native breed in Siberia with an ancestry tracing back to indigenous Siberian cattle that migrated 1000 years ago from the southern Baikal region (Y symbol in Fig. 3)^33^. However, a survey of 54 mtDNAs from this breed revealed only haplogroup T mtDNAs, similarly to Mongolian native cattle^33–35^. In China, a recent mtDNA survey of 24 aurochs remains from a large Neolithic (6300 to 5000 YBP) pit in northeast China (Houtaomuga ruins, Y symbol in Fig. 3) revealed haplogroups C (23 samples) and T (1 sample)^11^. Overall these analyses do not provide evidence of the presence of P mtDNAs either in Yakutian and Mongolian cattle or Chinese aurochs. It is also conceivable that the Asian continental source of P1a still exists but it is in a remote and not yet surveyed area of Central/Northeast Asia or even that such a source does not exist anymore, having been replaced by more productive non-autochthonous cattle breeds. In any cases, currently available mtDNA data from continental Asian cattle breeds and extinct aurochs are still inadequate for providing clearcut answers on how and when P1a entered the gene pool of modern Asian cattle.

However, there are data that provide some clues on the source and arrival time of P1a mtDNAs in Japan. Out of four Wagyu breeds, only the Japanese Shorthorn cattle harbor P1a. Unlike the other Japanese breeds whose ancestors derive from Korea (solid-line arrow in Fig. 3)^36^, the Japanese Shorthorn ancestors derive from a more northern continental source and followed a different entry route (red broken-line arrow in Fig. 3). Indeed, two historical documents dated to the sixteenth and seventeenth centuries report that hundreds of horse and cattle were imported from Mongolia and/or Russia to northern Japan in 1454–1456 A.D. (565 YBP) for military buildup^37^. Such an arrival time fits rather well with both the BSP data indicating a sharp increase in population size for P1a about 650 YBP (Fig. 2) and the star-like topology of P1a (Supplementary Fig. S3).

In conclusion, our survey of mitogenomes from Japanese Shorthorn cattle followed by combined analyses of all data concerning haplogroup P appear to indicate a gradual post-glacial expansion of aurochs from Europe to Asia, an early incorporation of aurochs cows into northeast Asian domestic herds, and a rather recent arrival of this haplogroup in Japan. It is clear that definitive answers concerning the source and the mode of arrival of haplogroup P in Asia require additional mitogenome data from modern autochthonous taurine breeds and especially ancient aurochs.

## Materials and methods

### Samples and mitogenome sequencing

DNAs from 14 Japanese Shorthorn cattle whose mtDNAs had been previously classified within haplogroup P by a survey of the control-region sequence variation were available^20^. Each of the 14 samples supposedly harbored one of 14 control-region haplotypes^20^. By following the approach previously described^22^, we were able to amplify two long-range overlapping PCR fragments encompassing the entire mitogenome. Through Next Generation Sequencing with an Illumina MiSeq by TaKaRa Bio Inc. (Kusatsu, Japan) and comparison with the bovine reference sequence (BRS, GenBank accession number V00654)^38^, we then identified a total of ten novel complete mitogenome haplotypes (JS1-JP10) in the 14 Japanese Shorthorn samples (Supplementary Fig. S1).

### Phylogenetic analyses and age estimates of haplogroup P nodes

For phylogenetic analyses, we used representative mitogenome sequences of haplogroups T1 (KT184462, KT184463, JN817321 - JN817329), T2 (KT184456, KT184457), T3 (V00654, KT184451, KT184452), T5 (EU177862), P (DQ124389, GU985279, JQ437479), Q1 (HQ184036, HQ184039, KT184471, KT184472, FJ971082, FJ971083), Q2 (FJ971081, HQ184030), R1 (FJ971084, FJ971085, HQ184040), R2 (FJ971087), I1 (NC005971) and I2 (EU177869). These sequences were aligned by using the MEGA package Ver. 7.0^39^. A Maximum Likelihood (ML) tree was constructed, and its confidence was assessed by the ultrafast bootstrap method, with 1000 replications incorporated into the IQ-tree ver. 1.6.12^40^. The nucleotide substitution model was selected based on the BIC, and the HKY + I model was selected as the best model. We also constructed additional ML phylogenetic trees using 410-bp long control-region sequences (from np 15,903 to np 16,312) from modern P1a mtDNAs and the P mtDNAs from European aurochs previously reported^8^.

Coalescent times were estimated based on the concatenated 13 protein-coding genes encoded in the mitogenome by using the BASEML program implemented in PAML ver. 4.1^41^ under the global clock model. Since only the *ND6* gene among the 13 protein-coding genes is encoded in the L strand, and considering the remarkable nucleotide composition bias of the mitogenome sequence, the complementary strand was used for *ND6*. Taking into account the different tempo and mode in nucleotide substitutions, the parameters of three codon positions were separately estimated. Since it is reported that the effect of the time dependency of the molecular evolutionary rate is reduced in third codon positions^42^, the results of the whole codon positions and the third codon position only were compared. The split time of *Bos taurus* and *Bos indicus* was assumed to be 335 thousand years ago^23^. Since the geological ages of terminal nodes cannot be taken into account in this method, results with inclusion and exclusion of aurochs were both assessed.

### Demographic analyses

Control-region sequences (410-bp long, from np 15,903 to np 16,312) belonging to haplogroup P mtDNAs from aurochs as well as modern cattle were used for demographic analyses (36 aurochs and 86 modern cattle). BEAST ver.1.10.4^43^ was used for Bayesian skyline plot (BSP)^44^, under the MCMC of 50 million runs. Trees were sampled every 1000 runs and the first 5 million runs were discarded as burn-in. The HKY + Γmodel was used for the nucleotide substitution model. Alignment data of aurochs and cattle were separately analyzed. As for aurochs data, the C14 dates of archaeological specimens were used for time calibrations. The mutation rate in control-region sequences was assumed to be uniform between aurochs and cattle, and the mutation rate estimates based on aurochs data were directly applied to cattle data to infer the demographic history of cattle.

### Ethics statement

All experiments were carried out according to the Kobe University Animal Experimentation Regulations, and the all protocols were approved by the Institutional Animal Care and Use Committee of Kobe University and by Association for the Promotion of Research Integrity (Approval Number: AP0000436777). All blood samples collections were approved by animal owners with signed informed consent and were collected by veterinarians or individual livestock owners in accordance with the Japanese Veterinarians Act (Act No. 186 of 1949).

## Supplementary information

Supplementary Information.

## Data Availability

The ten entire mitogenome sequences are deposited and available in DDBJ database (accession numbers: LC537308 - LC537317).
