# Supplementary material for: Cattle mitogenome variation reveals a post-glacial expansion of haplogroup P and an early incorporation into northeast Asian domestic herds
Source: Sci Rep. 2020 Nov 30;10:20842. doi: 10.1038/s41598-020-78040-8 (PMC7704668; doi:10.1038/s41598-020-78040-8)
Supplement: Supplementary file 1 — Supplementary Information. [file 41598_2020_78040_MOESM1_ESM.pdf]

Supplementary Information

Title: Cattle mitogenome variation reveals a post-glacial expansion of haplogroup P and an early incorporation into northeast Asian domestic herds

Hideyuki Mannen, Takahiro Yonezawa, Kako Murata, Aoi Noda, Fuki Kawaguchi, Shinji Sasazaki, Anna Olivieri, Alessandro Achilli,

& Antonio Torroni

Table S1. Coalescence times of cattle haplogroups estimated by ML method (years ago)

Node[1]	with Aurochs[2]						without Aurochs[2]					
	CDS[3]			3rd codon[3]			CDS			3rd codon		
Node 1	335,000			335,000			335,000			335,000		
Node 2	38,030	±	8,630	43,240	±	10,510	37,970	±	8,620	43,140	±	10,500
Node 3	137,420	±	15,520	144,100	±	17,810	136,550	±	15,520	143,470	±	17,820
Node 4	46,040	±	9,630	41,190	±	10,460	45,890	±	9,610	41,010	±	10,430
Node 5	4,160	±	2,440	1,720	±	1,910	4,150	±	2,440	1,710	±	1,900
Node 6	65,990	±	9,640	67,580	±	11,110	64,690	±	9,720	66,820	±	11,150
Node 7	16,180	±	4,560	8,770	±	3,650	NA[4]			NA		
Node 8	12,530	±	4,010	8,770	±	3,620	NA			NA		
Node 9	3,720	±	1,770	2,900	±	1,980	3,580	±	1,510	2,790	±	1,610
Node 10	47,000	±	7,950	47,950	±	9,100	16,570	±	3,380	12,990	±	3,510
Node 11	16,630	±	3,450	13,040	±	3,610	16,570	±	3,360	12,990	±	3,450
Node 12	16,630	±	3,390	13,040	±	3,520	16,570	±	3,370	12,990	±	3,450
Node 13	2,410	±	2,360	0	±	590	13,730	±	3,970	17,520	±	5,090
Node 14	17,250	±	4,520	21,670	±	5,760	8,420	±	3,050	10,820	±	3,920
Node 15	8,450	±	3,060	10,870	±	3,940	12,950	±	2,720	11,560	±	4,510
Node 16	12,990	±	2,820	11,610	±	4,610	12,950	±	2,660	11,560	±	4,500
Node 17	10,990	±	3,810	4,990	±	3,020	10,930	±	3,780	4,950	±	2,920

[1]Node numbers correspond to those in Fig. S2. Nodes 7, 8 and 9 correspond to P, P1 and P1a, respectively.

[2]with/without aurochs: analysis with/without mitogenome sequences of aurochs.

[3]Analysis using all substitutions in protein-coding regions or only 3rd codon substitutions.

[4]NA: not applicable.

a

region	D-loop																																
position	15951	15953	15994	16019	16049	16051	16058	16068	16074	16085	16119	16121	16122	16133	16141	16231	16247	16248	16255	16264	16301	106	166	169	173	190	221	222	249	300	301	349	363
Haplotype (accession Nos.) [N]																											i					d	d
T3 (V00654)	T	C	A	T	C	T	C	T	T	T	T	G	T	T	T	C	C	C	T	G	C	T	A	A	A	C	-	T	T	G	C	A	C
JS1 (LC537308)[4]	C	G	G	.	T	C	T	.	C	C	.	.	C	.	.	T	T	.	C	A	.	C	G	.	.	T	C	.	C	A	T	.	.
JS2 (LC537309) [1]	C	G	G	.	T	C	T	.	C	C	.	.	C	.	.	T	T	.	C	A	.	C	G	G	.	T	C	.	C	A	T	.	.
JS3 (LC537310)[2]	C	G	G	.	T	C	T	.	C	C	.	.	C	C	.	T	T	.	C	A	.	C	G	.	.	T	C	.	C	A	T	.	.
JS4 (LC537311) [1]	C	G	G	.	T	C	T	.	C	C	.	.	C	.	.	T	T	.	C	A	.	C	G	.	.	.	C	.	C	A	T	.	.
JS5 (LC537312) [1]	C	G	G	.	T	C	T	.	C	C	.	.	C	.	.	T	T	T	C	A	.	C	G	.	.	T	C	.	C	A	T	.	.
JS6 (LC537313) [1]	C	G	G	.	T	C	T	.	C	C	C	.	C	.	.	T	T	.	C	A	.	C	G	.	.	T	C	C	C	A	T	.	.
JS7 (LC537314) [1]	C	G	G	.	T	C	T	C	C	C	.	.	C	.	.	T	T	.	C	A	.	C	G	.	.	T	C	.	C	A	T	-	.
JS8 (LC537315) [1]	C	G	G	.	T	C	T	.	C	C	.	.	C	C	.	T	T	.	C	A	.	C	G	.	.	T	C	C	C	A	T	.	.
JS9 (LC537316) [1]	C	G	G	.	T	C	T	.	C	C	.	.	C	.	.	T	T	.	C	A	.	C	G	.	.	T	C	.	C	A	T	-	.
JS10 (LC537317) [1]	C	G	G	.	T	C	T	.	C	C	.	.	C	.	.	T	T	.	C	A	.	C	G	.	.	T	C	.	C	A	T	.	.
P (DQ124389)	C	G	G	.	T	C	T	.	C	C	.	.	C	.	.	T	T	.	C	A	.	C	G	.	G	T	C	C	C	A	T	.	.
P (GU985279)	C	G	G	C	T	C	T	.	C	C	.	R	C	.	C	T	.	.	C	A	T	.	.	.	.	T	C	.	C	A	T	.	-
P (JQ437479)	C	G	G	.	T	.	T	.	C	C	.	.	C	.	.	T	.	.	C	A	.	.	.	G	.	T	C	.	C	A	T	.	-

region	12S/rRNA		16S rRNA							ND1					tRNA/Gln	tRNA/Met	ND2				tRNA/Asn	tRNA/Tyr	CoI						tRNA/Asp	CoII			ATPase8	ATPase6		CoIII	ND3
position	587	1128	1481	2145	2171	2536	2558	2585	2711	3118	3302	3379	3550	3694	4150	4252	4293	4676	5156	5259	5501	5681	5743	5890	5899	6160	6436	6451	7356	7604	7952	7994	8236	8358	8370	9682	10126
Haplotype (accession Nos.) [N]	i																																				
T3 (V00654)	-	A	G	G	G	C	G	T	G	T	G	T	G	T	C	T	C	A	G	T	C	T	T	C	A	T	G	T	A	T	C	A	T	C	A	G	C
JS1 (LC537308)[4]	C	G	A	.	A	A	A	C	.	.	.	C	A	.	.	C	T	G	A	.	T	C	C	T	G	C	A	.	G	C	T	G	C	T	C	C	T
JS2 (LC537309) [1]	C	G	A	.	A	A	A	C	.	.	.	C	A	.	.	C	T	G	A	.	T	C	C	T	G	C	A	.	G	.	T	G	C	T	C	C	T
JS3 (LC537310)[2]	C	G	A	.	A	A	A	C	.	.	.	C	A	.	.	C	T	G	A	.	T	C	C	T	G	C	A	.	G	.	T	G	C	T	C	C	T
JS4 (LC537311) [1]	C	G	A	.	A	A	A	C	.	.	.	C	A	.	.	C	T	G	A	.	T	C	C	T	G	C	A	.	G	.	T	G	C	T	C	C	T
JS5 (LC537312) [1]	C	G	A	.	A	A	A	C	.	.	A	C	A	.	T	C	T	G	A	.	T	C	C	T	G	C	A	.	G	.	T	G	C	T	C	C	T
JS6 (LC537313) [1]	C	G	A	.	A	A	A	C	.	.	.	C	A	.	.	C	T	G	A	C	T	C	C	T	G	C	A	.	G	.	T	G	C	T	C	C	T
JS7 (LC537314) [1]	C	G	A	.	A	A	A	C	A	.	.	C	A	C	.	C	T	G	A	.	T	C	C	T	G	C	A	.	G	.	T	G	C	T	C	C	T
JS8 (LC537315) [1]	C	G	A	.	A	A	A	C	.	.	.	C	A	.	.	C	T	G	A	.	T	C	C	T	G	C	A	.	G	.	T	G	C	T	C	C	T
JS9 (LC537316) [1]	C	G	A	.	A	A	A	C	.	.	.	C	A	C	.	C	T	G	A	.	T	C	C	T	G	C	A	.	G	.	T	G	C	T	C	C	T
JS10 (LC537317) [1]	C	G	A	.	A	A	A	C	.	.	.	C	A	.	.	C	T	G	A	.	T	C	C	T	G	C	A	.	G	.	T	G	C	T	C	C	T
P (DQ124389)	C	G	A	.	A	A	A	C	.	.	.	C	A	.	.	C	T	G	A	.	T	C	C	T	G	C	A	.	G	.	T	G	C	T	C	C	T
P (GU985279)	C	G	A	A	.	A	A	C	.	.	.	C	A	.	.	C	T	G	A	.	T	.	C	T	G	C	A	.	G	.	T	G	C	T	C	C	T
P (JQ437479)	C	G	A	.	.	A	A	C	.	C	A	C	A	.	.	.	.	G	A	.	T	.	C	T	G	C	A	C	G	.	T	G	C	T	C	C	T

region	ND4						tRNA/Ser	ND5													ND6		Cytb					tRNA/Thr	
position	10691	11000	11140	11468	11740	11791	12016	12246	12377	12433	12468	12469	12525	12675	12738	12750	13005	13310	13539	13821	14036	14129	14580	14873	15134	15471	15627	15673	15714
Haplotype (accession Nos.) [N]																													
T3 (V00654)	C	C	A	G	C	C	T	C	T	T	T	C	G	C	C	T	A	A	T	A	G	G	G	G	C	C	A	T	C
JS1 (LC537308)[4]	G	T	G	A	.	.	C	.	C	C	C	.	.	T	T	C	G	C	.	G	A	T	.	A	T	.	G	C	T
JS2 (LC537309) [1]	G	T	G	A	.	.	C	.	C	C	C	.	.	T	T	C	G	C	.	G	A	T	.	A	T	.	G	C	T
JS3 (LC537310)[2]	G	T	G	A	.	.	C	T	C	C	C	.	.	T	T	C	G	C	.	G	A	T	.	A	T	.	G	C	T
JS4 (LC537311) [1]	G	T	G	A	.	T	C	.	C	C	C	.	.	T	T	C	G	C	.	G	A	T	.	A	T	.	G	C	T
JS5 (LC537312) [1]	G	T	G	A	.	.	C	.	C	C	C	.	.	T	T	C	G	C	.	G	A	T	.	A	T	.	G	C	T
JS6 (LC537313) [1]	G	T	G	A	.	.	C	.	C	C	C	.	.	T	T	C	G	C	.	G	A	T	.	A	T	.	G	C	T
JS7 (LC537314) [1]	G	T	G	A	.	.	C	.	C	C	C	.	.	T	T	C	G	C	.	G	A	T	.	A	T	.	G	C	T
JS8 (LC537315) [1]	G	T	G	A	.	.	C	T	C	C	C	.	.	T	T	C	G	C	.	G	A	T	.	A	T	.	G	C	T
JS9 (LC537316) [1]	G	T	G	A	.	.	C	.	C	C	C	.	.	T	T	C	G	C	.	G	A	T	.	A	T	.	G	C	T
JS10 (LC537317) [1]	G	T	G	A	.	T	C	.	C	C	C	.	.	T	T	C	G	C	.	G	A	T	.	A	T	.	G	C	T
P (DQ124389)	G	T	G	A	.	.	C	.	C	.	C	.	.	T	T	C	G	C	.	G	A	T	.	A	T	.	.	C	Y
P (GU985279)	G	T	G	.	T	.	C	.	C	C	C	T	A	T	.	C	G	C	.	G	A	T	A	A	T	.	G	C	.
P (JQ437479)	G	T	G	.	.	.	C	.	C	C	C	.	.	T	.	C	G	C	C	G	A	T	.	A	T	T	G	C	.

b

position	16019	16051	16068	16119	16121	16133	16141	16247	16248	16301	106	166	169	173	190	222	349	363	2145	2171	2711	3118	3302	3694	4150	4252	4293	5259	5681	6451	7604	11468	11740	11791	12246	12433	12469	12525	12738	13539	14580	15471	15627	15714
Haplotype (accession Nos.) [N]																	d	d																										
T3(V00654)	T	T	T	T	G	T	T	C	C	C	T	A	A	A	C	T	A	C	G	G	G	T	G	T	C	T	C	T	T	T	T	G	C	C	C	T	C	G	C	T	G	C	A	C
JS1 (LC537308)[4]	.	C	.	.	.	.	.	T	.	.	C	G	.	.	T	.	.	.	.	A	.	.	.	.	.	C	T	.	C	.	C	A	.	.	.	C	.	.	T	.	.	.	G	T
JS2 (LC537309)	.	C	.	.	.	.	.	T	.	.	C	G	G	.	T	.	.	.	.	A	.	.	.	.	.	C	T	.	C	.	.	A	.	.	.	C	.	.	T	.	.	.	G	T
JS3 (LC537310)[2]	.	C	.	.	.	C	.	T	.	.	C	G	.	.	T	.	.	.	.	A	.	.	.	.	.	C	T	.	C	.	.	A	.	.	T	C	.	.	T	.	.	.	G	T
JS4 (LC537311)	.	C	.	.	.	.	.	T	.	.	C	G	.	.	.	.	.	.	.	A	.	.	.	.	.	C	T	.	C	.	.	A	.	T	.	C	.	.	T	.	.	.	G	T
JS5 (LC537312)	.	C	.	.	.	.	.	T	T	.	C	G	.	.	T	.	.	.	.	A	.	.	A	.	T	C	T	.	C	.	.	A	.	.	.	C	.	.	T	.	.	.	G	T
JS6 (LC537313)	.	C	.	C	.	.	.	T	.	.	C	G	.	.	T	C	.	.	.	A	.	.	.	.	.	C	T	C	C	.	.	A	.	.	.	C	.	.	T	.	.	.	G	T
JS7 (LC537314)	.	C	C	.	.	.	.	T	.	.	C	G	.	.	T	.	-	.	.	A	A	.	.	C	.	C	T	.	C	.	.	A	.	.	.	C	.	.	T	.	.	.	G	T
JS8 (LC537315)	.	C	.	.	.	C	.	T	.	.	C	G	.	.	T	C	.	.	.	A	.	.	.	.	.	C	T	.	C	.	.	A	.	.	T	C	.	.	T	.	.	.	G	T
JS9 (LC537316)	.	C	.	.	.	.	.	T	.	.	C	G	.	.	T	.	-	.	.	A	.	.	.	C	.	C	T	.	C	.	.	A	.	.	.	C	.	.	T	.	.	.	G	T
JS10 (LC537317)	.	C	.	.	.	.	.	T	.	.	C	G	.	.	T	.	.	.	.	A	.	.	.	.	.	C	T	.	C	.	.	A	.	T	.	C	.	.	T	.	.	.	G	T
P (DQ124389)	.	C	.	.	.	.	.	T	.	.	C	G	.	G	T	C	.	.	.	A	.	.	.	.	.	C	T	.	C	.	.	A	.	.	.	.	.	.	T	.	.	.	.	Y
P (GU985279)	C	C	.	.	R	.	C	.	.	T	.	.	.	.	T	.	.	-	A	.	.	.	.	.	.	C	T	.	.	.	.	.	T	.	.	C	T	A	.	.	A	.	G	.
P (JQ437479)	.	.	.	.	.	.	.	.	.	.	.	.	G	.	T	.	.	-	.	.	.	C	A	.	.	.	.	.	.	C	.	.	.	.	.	C	.	.	.	C	.	T	G	.

Supplementary Figure S1. Sequence variation observed in entire *Bos taurus* mitogenomes belonging to haplogroup P. a: Sequence alignment of the ten P haplotypes (JS1-JS10) identified in Japanese Shorthorn cattle and the three previously published (DQ124389, GU985279 and JQ437479) in comparison with the bovine reference sequence (V00654) – a member of haplogroup T3. JS1 and JS3 were detected in four and two subjects, respectively. b: Sequence alignment within haplogroup P. An "i" indicates

an insertion, a “d” indicates a deletion. In this study, we carried out, by Next Generation Sequencing (NGS) and using an Illumina MiSeq, entire mitogenome sequencing for 14 samples, one each for the 14 control-region haplotypes previously detected. However, with the new sequencing approach, we were able to identify only ten haplotypes (JS1-JS10) (DDBJ accession numbers: LC537308 - LC537317) in the 14 samples, due to incorrect assignment of the variation obtained by Sanger sequencing in the previous study [20]. The sequences obtained in the previous study were thus corrected and renewed (Accession numbers: LC314264 - LC314271).

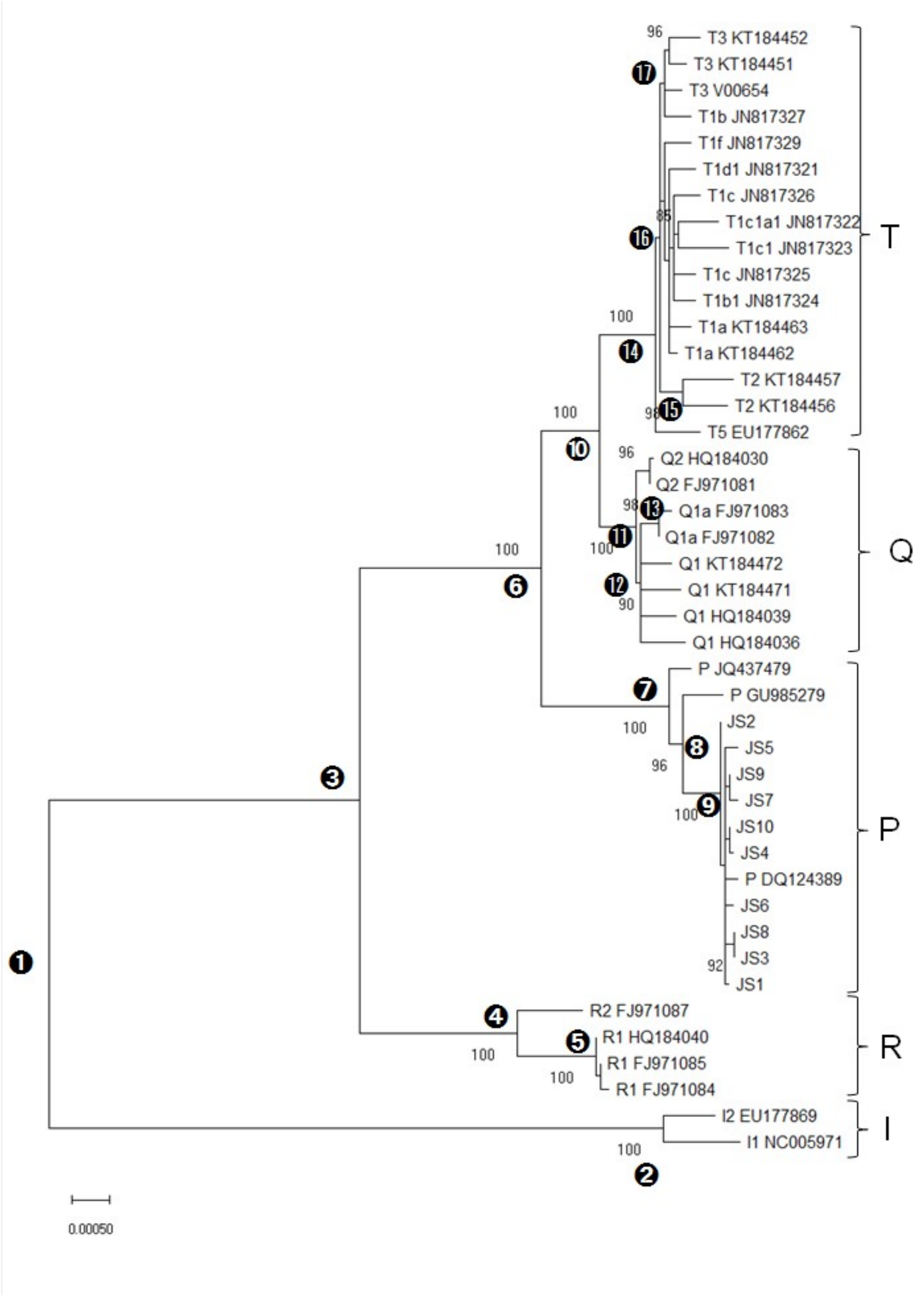

Supplementary Figure S2. **Maximum Likelihood (ML) tree encompassing haplogroup P mitogenomes (13 haplotypes in 18 specimens) as well as representative mitogenomes from other haplogroups.** Sequence alignment was performed using the MEGA package Ver. 7.0 [39]. The phylogenetic tree was constructed by using representative sequences of haplogroups T1, T2, T3, T5, Q1, Q2, R1, R2, I1 and I2, together with all P mitogenomes analyzed in this study. The confidence of the tree was assessed by the bootstrap method of MEGA 7.0, with 1000 replications. Numbers within black circles mark nodes whose coalescence times are provided in Table S1. Scale bar indicates genetic distance.

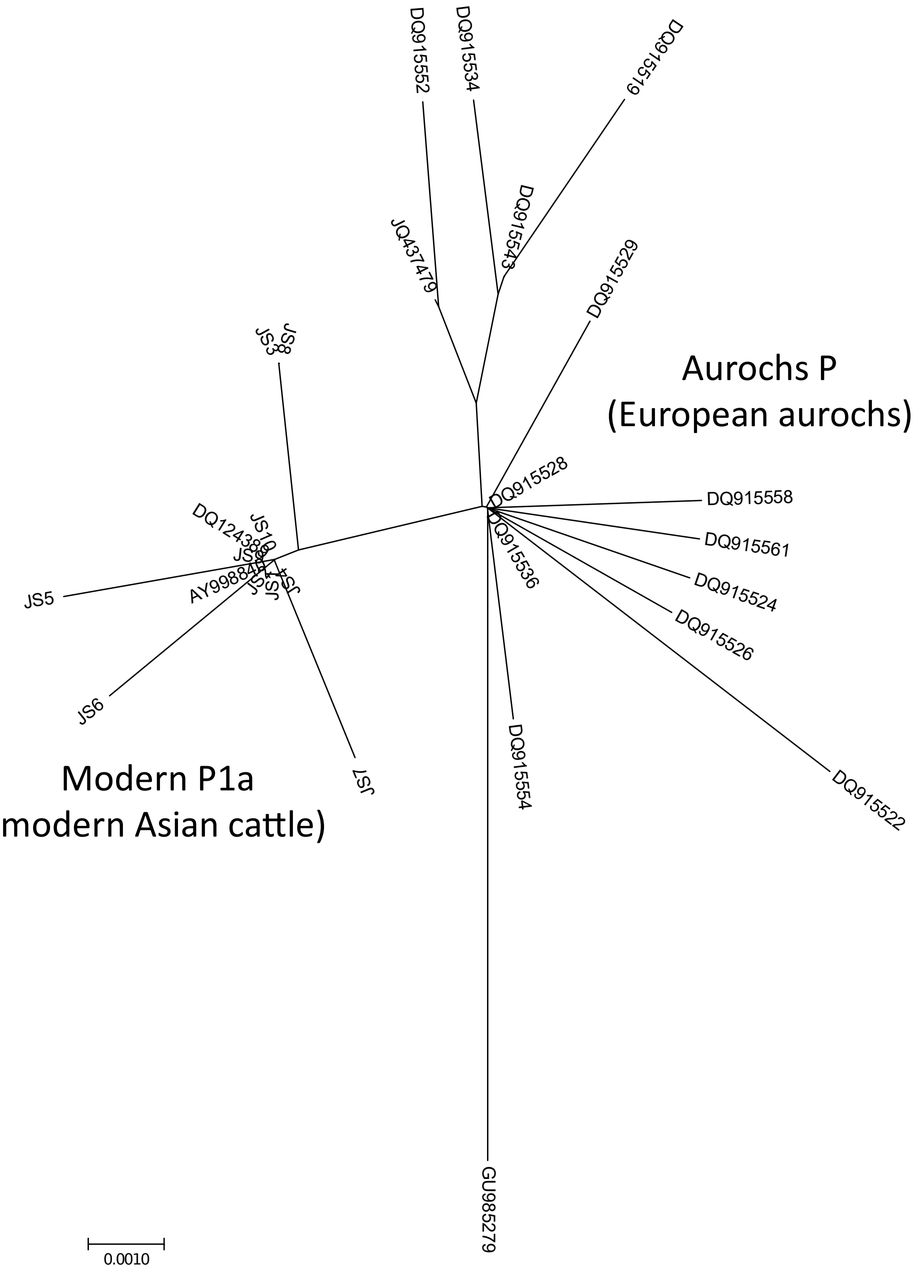

Supplementary Figure S3. ML tree obtained by using control-region sequences (410-bp) from haplogroup P mtDNAs. Sequences JS1-S10 were obtained in this study. Sequences from Korea (DQ124389) and China (AY998840) are identical to the JS1 sequence. The other P sequences are from the literature [8,20]. Scale bar indicates genetic distance.
